# Stomatal cell wall composition: distinctive structural patterns associated with different phylogenetic groups

**DOI:** 10.1093/aob/mcw275

**Published:** 2017-01-31

**Authors:** Ilana Shtein, Yaniv Shelef, Ziv Marom, Einat Zelinger, Amnon Schwartz, Zoë A. Popper, Benny Bar-On, Smadar Harpaz-Saad

**Affiliations:** 1 The Robert H. Smith Institute of Plant Sciences and Genetics in Agriculture, The Hebrew University of Jerusalem, Rehovot 7610001, Israel; 2 Department of Mechanical Engineering, Ben-Gurion University of the Negev, Beer Sheva 84105, Israel; 3 The Interdepartmental Equipment Unit, The Robert H. Smith Faculty of Agriculture, Food & Environment, The Hebrew University of Jerusalem, Rehovot 7610001, Israel; 4 Botany and Plant Science, Ryan Institute for Environmental, Marine and Energy Research, School of Natural Sciences, National University of Ireland Galway, Galway, Ireland

**Keywords:** Stomata, guard cells, cell wall, biomechanics, cellulose crystallinity, lignin, angiosperms, ferns, grasses

## Abstract

**Background and Aims** Stomatal morphology and function have remained largely conserved throughout ∼400 million years of plant evolution. However, plant cell wall composition has evolved and changed. Here stomatal cell wall composition was investigated in different vascular plant groups in attempt to understand their possible effect on stomatal function.

**Methods** A renewed look at stomatal cell walls was attempted utilizing digitalized polar microscopy, confocal microscopy, histology and a numerical finite-elements simulation. The six species of vascular plants chosen for this study cover a broad structural, ecophysiological and evolutionary spectrum: ferns (*Asplenium nidus* and *Platycerium bifurcatum*) and angiosperms (*Arabidopsis thaliana* and *Commelina erecta*) with kidney-shaped stomata, and grasses (angiosperms, family Poaceae) with dumbbell-shaped stomata (*Sorghum bicolor* and *Triticum aestivum*).

**Key Results** Three distinct patterns of cellulose crystallinity in stomatal cell walls were observed: Type I (kidney-shaped stomata, ferns), Type II (kidney-shaped stomata, angiosperms) and Type III (dumbbell-shaped stomata, grasses). The different stomatal cell wall attributes investigated (cellulose crystallinity, pectins, lignin, phenolics) exhibited taxon-specific patterns, with reciprocal substitution of structural elements in the end-walls of kidney-shaped stomata. According to a numerical bio-mechanical model, the end walls of kidney-shaped stomata develop the highest stresses during opening.

**Conclusions** The data presented demonstrate for the first time the existence of distinct spatial patterns of varying cellulose crystallinity in guard cell walls. It is also highly intriguing that in angiosperms crystalline cellulose appears to have replaced lignin that occurs in the stomatal end-walls of ferns serving a similar wall strengthening function. Such taxon-specific spatial patterns of cell wall components could imply different biomechanical functions, which in turn could be a consequence of differences in environmental selection along the course of plant evolution.

## INTRODUCTION

Stomata evolved in the late Silurian to early Devonian (Edwards *et al.*, 1986, 1998) and are one of the key innovations in plant evolution. According to Edwards *et al.* (1998), stomatal structure is the most conserved of land plant vegetative characters, presenting similar morphology and architecture throughout ∼400 million years of plant evolution. In extant plants, the earliest stomata are found in the Bryophyta (but seen only in the spermatophyte phase) (Ligrone *et al.*, 2012). All vascular plants have abundant stomata (Ziegler, 1987), while the most structurally advanced and efficient stomata are the dumbbell-shaped stomata of grasses. However, the acquisition of stomatal regulation is currently subject to debate, with some authors considering that regulation was attained early in stomatal evolution while others consider that stomatal regulation was more gradually acquired (Duckett *et al.*, 2009; Chater *et al.*, 2011, 2015; Ruszala *et al.*, 2011; McAdam and Brodribb, 2012, 2013, 2015; Pressel *et al.*, 2014; Merced and Renzaglia, 2016). Regardless of whether stomatal function was acquired early in stomatal evolution, or gradually, the stomata of extant Bryophyta have a role in gas exchange even if the primary role of the earliest stomata was as drying pores, i.e. aiding water loss from the sporophyte leading to spore release (Duckett *et al.*, 2009; Merced and Renzaglia, 2013, 2014; Merced, 2015). In all vascular plant species, stomatal cell walls undergo reversible deformation during opening/closing of the pore, requiring an extremely strong and flexible structure.

Plant cell walls are composed of a complex and heterogeneous polysaccharide network dictating the mechanical properties of the walls including tensile and compressive properties and a range of flexibilities (Doblin *et al.*, 2010). It is known that distinct phylogenetic differences occurred in the cell wall constituents of a wide range of plants including bryophytes, mosses, ferns and seed plants (Popper and Fry, 2003, 2004). Although all extant plants have a relatively conserved stomatal morphology, several broad evolutionary changes have occurred in stomatal cell wall structure and composition. For instance, ferns and gymnosperms often have lignified guard cell walls (Ziegler, 1987). In the Equisetaceae guard cell walls are partially silicified and have overlapping subsidiary cells with unique thickenings (Ziegler, 1987). Particularly noteworthy are the stomata of grasses, which are dumbbell-shaped and have superior stomatal control. While guard cell wall strength is generated by thick, sometimes lignified or silicified, walls, stomatal flexibility is achieved, at least in some species, by pectic materials. For example, arabinans have an important function in stomatal opening in *Commelina* (Jones *et al.*, 2003). Interestingly, three plants from different angiosperm families were shown to deposit abundant pectins and phenolic esters in their guard cell walls, even grass species whose cell walls characteristically have low levels of pectins (Jones *et al.*, 2005). Together this implies that pectins might have a conserved functional role in stomata and that modification in guard cell wall composition may affect stomata function.

In plant cell walls, stiff cellulose microfibrils (macromolecular structures composed of arrays of β-1,4 glucan chains, associated through numerous hydrogen bonds) are embedded in a gel-like matrix of non-cellulosic polysaccharides. The cellulose microfibrils can vary in composition over their length, with regions exhibiting differing degrees of crystallinity, depending on the properties of the lower structural order, i.e. the elementary fibrils (Klemm *et al.*, 2005). The degree of cellulose crystallinity affects both the chemical and the mechanical properties of the cell wall, such as the ability to bind water and other cell wall polysaccharides (Goto and Yokoe, 1996; Chami Khazraji and Robert, 2013; Gu and Catchmark, 2014; Park and Cosgrove, 2015). Due to strong differences in stiffness between the cellulose microfibrils and the other cell wall polymers, the orientation of microfibrils predominantly dictates various biomechanical properties, including the direction of cellular expansion or shrinkage (Burgert and Fratzl, 2009). Even by the first half of the 20th century, using polar microscopy, Ziegenspeck (1938*a*, *b*) discovered a pivotal feature of the unique stomatal cell wall structure: in kidney-shaped stomata the cellulose microfibrils are radially orientated, while in the dumbbell-shaped stomata of grasses the microfibrils are arranged parallel to the pore (Ziegenspeck, 1938*a*, *b*; Palevitz, 1981). Accordingly, using simple sausage-shaped balloon models, Aylor *et al.* (1973) identified two structural features, or physical constraints, crucial for the opening mechanism that causes the guard cells to bend and thus open the stomatal pore. Those features were: (1) the particular arrangement of the cellulose microfibrils and (2) the constrained length of the stomatal apparatus. Microfibrils restrict movement in the radial direction and cause the guard cells to expand longitudinally, change shape and bend. Thus, the guard cell can be compared to a steel-belted radial tire (Sharpe *et al.*, 1987). In the dumbbell-shaped stomata of grasses, although the microfibrils have a parallel arrangement pattern, the microfibrils are arranged radially at the bulbous ends; the stoma opens by expansion and bending similarly to kidney-shaped stomata (Aylor *et al.*, 1973). However, the model proposed by Aylor *et al.* disregarded both the cell wall morphology and the actual increase in cell volume. Several other models exist (e.g. DeMichele and Sharpe, 1973; Cooke *et al.*, 1976), predominantly dating to ∼40 years ago, and each having a number of shortcomings. Although there is a vast body of contemporary research focused on stomata, no biomechanical models have been constructed and published in recent decades, despite significant progress in the field of biomechanics.

Stomata offer a unique research system, where morphology and function have predominantly remained the same, even though cell wall features of the various plant groups are known to have evolved and changed. Recently, a combined genetics and cell biology approach has been applied to investigate the role of cellulose, as well as the hemicellulose xyloglucan, in stomatal guard cell movement in *Arabidopsis thaliana* (Rui and Anderson, 2016). The data presented suggest that both cellulose and xyloglucan are required for proper stomata function and that cellulose is reorganized, from evenly spaced to bundled microfibrils, during stomata movement (Rui and Anderson, 2016). In the current research we attempted a renewed look at stomatal cell wall structure, utilizing computerized polarized light microscopy (PolScope) and confocal microscopy, in six plant species representing a broad structural and evolutionary spectrum. 

## MATERIALS AND METHODS

### Plant material

*Arabidopsis thaliana* (L.) Heynh. (ecotype Columbia, Col-0) plants were grown at 22 °C in short-day (10 h/14 h light/dark) growth chambers and harvested at a fully mature stage after 45 d. *Triticum aestivum* (L.), *Sorghum bicolor* (L.) Moench and *Commelina erecta* (L.) leaves were obtained from the Faculty of Agriculture, Rehovot. *Asplenium nidus* (L.) was purchased at a local plant nursery and further grown in the lab. *Platycerium bifurcatum* (Cav.) C. Chr. fronds (megaphylls whose homology with angiosperm leaves is subject to debate; Vasco *et al.*, 2013) were obtained from the Tel Aviv University botanical garden. In *P. bifurcatum*, which has two frond types, only the antler fronds were used.

For all species only the fully expanded mature leaves were used. All experiments, except confocal microscopy, were made on epidermal peels obtained from the lower (abaxial) side of the leaf. Typically, the abaxial side of the leaf has more stomata and in many species the upper (adaxial) side is astomatous. Thus, to enable a more uniform comparison between the species we used only the abaxial side of the leaves. When epidermal peels were difficult to make, very thin paradermal sections were prepared by hand using a razor blade.

### Polarized light microscopy

Crystalline cellulose is a strongly birefringent material. The LC-PolScope image processing system (CRi, Inc., Woburn, MA, USA) with liquid crystal (LC) compensator enables the *in-situ* assessment of the sample light retardance, from which cellulose crystallinity and orientation can be measured (Abraham and Elbaum, 2013). PolScope was mounted on a microscope (Eclipse 80i, Nikon, Tokyo, Japan) using Plan Fluor ×40/0·75 OFN25 DIC M/N2 objective equipped with a cooled CCD camera. The images were taken with a different retardance range for each species, as specified in the scale of each image. Background retardance was always subtracted under the same conditions. The stomata were observed on fresh epidermal peels from the abaxial side of fully expanded mature leaves. Several leaves of numerous species were observed in preliminary experiments, in order to calibrate the measuring conditions. Stomatal focus for measurements was chosen by comparing visible light, orientation and retardance images and choosing only correctly aligned and focused stomata. Retardance values were extracted manually using Abrio 2.2 software (CRi, Inc.). Ten stomata and ten epidermal cells from at least three different plants were measured per species. As a crystalline cellulose control, we used Avicel (microcrystalline cellulose powder, Sigma, St Louis, MO, USA). The retardance of ten Avicel particles was measured under the same conditions as the stomata.

### Scanning electron microscopy (SEM)

Small leaf fragments from a middle leaf portion were excised and fixed using the methanol method as described by Talbot and White (2013). The samples were then critical-point-dried in a critical point dryer (CPD-030, Bal-Tec/Leica, Wetzlar, Germany) and gold coated in a gold sputter coating unit (Quorum Technologies/Polaron, Laughton, UK). The samples were observed by low-vacuum scanning electron microscopy (SEM; JSM 5410 LV, Jeol Ltd, USA). The SEM images were used to measure stomatal area.

### Fluorescence confocal microscopy

This experiment was performed on leaf fragments only (not on peels). Leaves were excised and immediately transferred to 95 % (v/v) ethanol for 2 d to optimally extract the chlorophyll. The leaves were then transferred to 70 % (v/v) ethanol. Small leaf fragments were transferred to double distilled water (DDW) with 0·02 % (v/v) Tween for rehydration overnight and then used for imaging.

Confocal laser scanning microscopy was performed on an inverted Leica sp8 confocal microscope with argon-ion and two He-Ne lasers. Excitation and detection windows were set as follows: Ex- 405, Em- 490–550 for lignin, and Em- 670–730 for chlorophyll. Laser power was set to 10 %. Stacks of 7–17 confocal optical sections (depending on species) were taken for lignin autofluorescence. All the images were taken using the same settings. Bright light images were taken in parallel (Supplementary Data Fig. S3a, b).

Images were handled using Fiji (FIJI Is Just ImageJ) software (Schindelin *et al.*, 2012). The lignin autofluorescence images were digitally coloured blue; autofluorescence derived from chlorophyll was coloured red.

### Phloroglucinol staining of lignin

Phloroglucinol stains the hydroxycinnamyl aldehyde end-groups in lignins (Kim *et al.*, 2002). A saturated solution of phloroglucinol (Sigma-Aldrich) was prepared in 18 % (v/v) HCl. Stomatal peels (prepared from leaf fragments pretreated as for fluorescence confocal microscopy) were stained with phloroglucinol for 5 min and viewed immediately. The stained sections were compared to unstained controls (Supplementary Data Fig. S3c, d). The samples were viewed and micrographed on and EVOS™ XL Core inverted microscope imaging system. The samples from different species were viewed at the same session using the same settings

### Ruthenium red (RR) staining of pectin

Leaves were excised and immediately transferred to 95 % (v/v) ethanol for 2 d, until the chlorophyll had been extracted. The leaves were then transferred to 70 % (v/v) ethanol. Large leaf fragments were transferred to DDW with 0·02 % (v/v) Tween 20 for 2 h to rehydrate and epidermal peels were prepared. Pectic substances were stained with 0·01 % (w/v) aqueous RR (Sigma-Aldrich) for 30 min. The samples were viewed and micrographed on an EVOS™ XL Core inverted microscope imaging system. The samples from different species were viewed at the same session using the same settings

### Modelling finite-elements simulation

A simplified stoma structure model for the numerical simulations was adapted from Sharpe and Wu (1978), in which the stoma structure is viewed as a curved cylinder with an elliptical inner contour (the stoma pore). On the material level, the stoma cellulose microfibrils were defined as locally aligned in the circumferential direction (see Fig. 9A), with an anisotropic stiffness ratio of 1:5 between the local microfibril direction and the orthogonal directions (see details in Supplementary Data and Gibson, 2012). Fixed boundary conditions were assumed for the stoma edges and a uniform pressure was assumed within the stoma (Fig. 9B). The stomata geometry was realized (SolidWorks, 2014, SolidWorks Corporation, Concord, MA, USA) and implemented into commercial finite-element simulation software (Abaqus 6.14, Simulia, Providence, RI, USA) in which the mechanical anisotropy of the stoma material was defined.

## RESULTS

### General observations

**Fig. 1.**
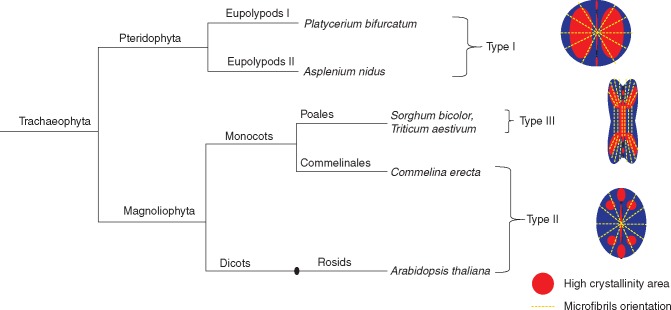
Phylogenetic tree of the species used for the current research. See main text for details on the schematic stomatal crystallinity types.

Stomata of the six species chosen for this research cover a broad structural and evolutionary spectrum (see Table 1 and Fig. 1). The species include two ferns from separate clades – *Platycerium bifurcatum* (Eupolypods I) and *Asplenium nidus* (Eupolypods II), which although they both possess large, kidney-shaped stomata belong to different photosynthetic types (CAM and C_3_, respectively); two angiosperm species with kidney-shaped stomata – *Arabidopsis thaliana* (eudicot) and *Commelina erecta* (monocot, order Commelinales); and two grass species (monocot, order Poales, family Poaceae) with dumbbell-shaped stomata belonging to different photosynthetic types – *Sorghum bicolor* (C_4_ photosynthesis) and *Triticum aestivum* (C_3_ photosynthesis).

**Table 1:** The representative species, family, habitat and their stomatal attributes*

Species	Family	Habitat	Photosynthesis type	Stomata shape	Stomatal control	Stomatal movement^a^	Stomatal area (µm^2^) (mean ± s.e.; *n* = 10)
*Platycerium bifurcatum*	Polypodiaceae	Epiphyte fern; rain forests	CAM^b^	Kidney	Passive^e^	Up-down	1368 ± 57
*Asplenium nidus*	Aspleniaceae	Either epiphyte or terrestrial fern; grows in shady, humid areas	C_3_^c^	Kidney	Passive^e^	Up-down	2384 ± 84
*Arabidopsis thaliana*	Brassicaceae	Annual weed; native to Europe, Asia and north-western Africa	C_3_	Kidney	Active	Sidewards, up-down	239 ± 19
*Commelina erecta*	Commelinaceae	Perennial herb; distributed worldwide, requires moisture	C_3_	Kidney	Active	Sidewards, up-down	710 ± 18
*Sorghum bicolor*	Poaceae	Grass; hot, dry regions, high irradiation, fields	C_4_^d^	Dumb-bell	Active, superior^f^	Sidewards, up-down	202 ± 8
*Triticum aestivum*	Poaceae	Grass; sunny fields, wide distribution	C_3_	Dumb-bell	Active, superior^f^	Sidewards, up-down	760 ± 74

* Data from:

*a*, Beerling and Franks (2009);

*b*, Rut *et al.* (2008);

*c*, Reinert (1998);

*d*, Giussani *et al.* (2001);

*e*, Brodribb and McAdam (2011);

*f*, Franks and Farquhar (2006).

The chosen plants varied in their epidermal morphology and the stomatal complex (guard cells together with their surrounding neighbour/subsidiary cells) structure (Fig. 3, Supplementary Data Fig. S1). Ferns had round, kidney-shaped stomata with the largest stomatal area among the species (Table 1, Fig. 3A, C). *Asplenium* had an anomocytic stomatal complex (having an irregular number of subsidiary cells without a distinguished appearance) whereas *Platycerium* stomata were copolocytic (i.e. the stoma is encircled by a U-shaped subsidiary cell with a second subsidiary cell encircling the first) and the epidermis is covered in relatively large star-shaped trichomes. Although *Arabidopsis* and *Commelina* both had common kidney-shaped stomata, *Arabidopsis* had small stomata, with guard cells positioned between pavement cells of the epidermis, lacking true subsidiary cells (Figs 3E and S1), whereas *Commelina* had a large stomatal complex with six subsidiary cells (Figs 3G and S1). Its epidermal cells contained numerous crystals that became birefringent under polarized light (Figs 3H and4D). *Sorghum* and *Triticum* had typical paracytic grass stomatal complexes, with dumbbell-shaped guard cells and two subsidiary cells parallel to the long axis of the guard cells (Figs 3I, K and S1). The *Sorghum* epidermis had characteristic cork cells and silica cells. Guard cells of all six species had inner wall thickenings, while *Arabidopsis* and *Commelina* had extremely thick ones. It is known that the most morphologically distinctive guard cell feature is their characteristic shape and non-uniform cell wall thickenings (Esau, 1965). The pattern of wall thickenings varies between species, although usually the upper and lower paradermal walls near the pore are thickened (Palevitz, 1981).

### Cellulose microfibril orientation in stomatal complex

**Fig. 2.**
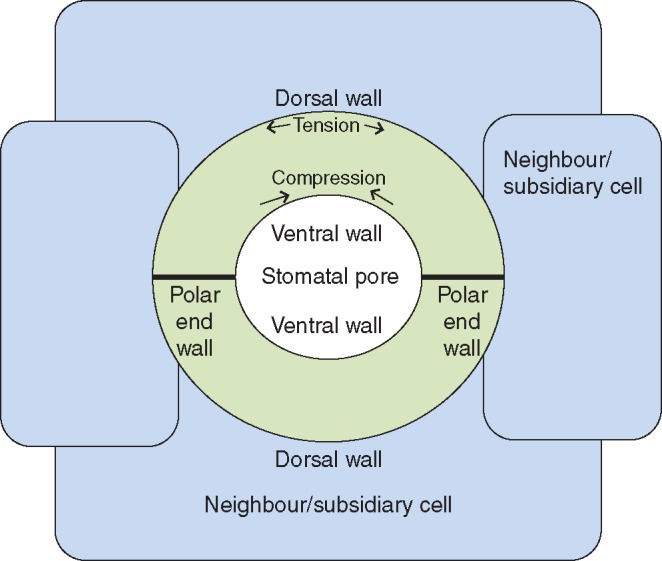
Schematic representation of a stomatal complex.

**Fig. 3.**
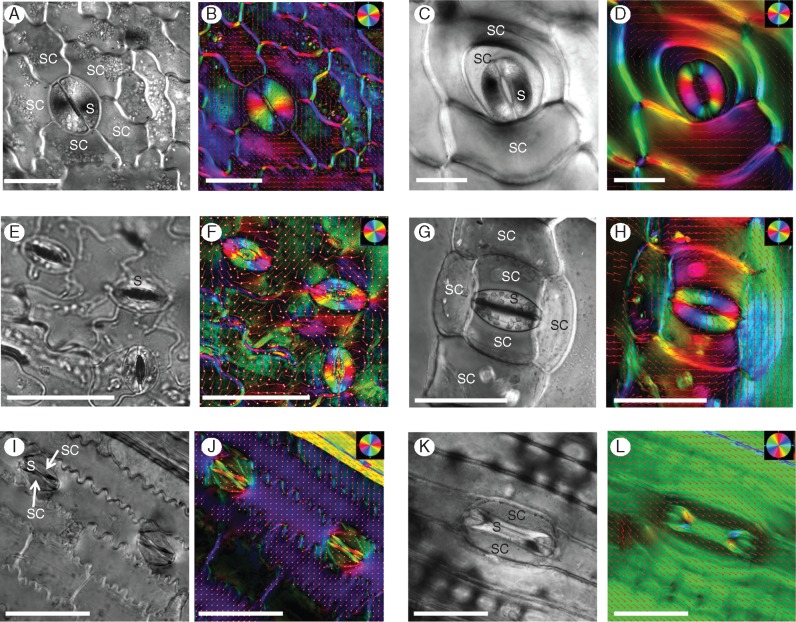
PolScope images of stomata showing crystalline cellulose orientation. Representative polarized light (left) and colour-coded images (right) of cellulose microfibril orientation are presented for each species. (A, B) *Asplenium*, (C, D) *Platycerium*, (E, F) *Arabidopsis*, (G, H) *Commelina* (note the birefringent crystals in the epidermis), (I, J) *Sorghum*, (K, L) *Triticum*. The orientation colour pie-chart codes the cellulose microfibril orientation for every image. The red vector arrows also show the orientation direction for a clearer view. S, stoma; SC, subsidiary cell. Size bars = 50 μm.

Initially, we observed the orientation of cellulose microfibrils in the stomata (see Fig. 2 for a schematic representation of the stomatal complex and interpretation of the cell wall structure). The orientation of cellulose microfibrils in the guard cells was consistent with what is known from the literature (Ziegler, 1987). The orientation was radial in the kidney-shaped stomata, both in ferns and in angiosperms (Fig. 3A–H) whereas in grasses, the microfibrils were orientated parallel in the narrow area and radially in the bulbous polar ends (Fig. 3I–L). The LC-PolScope enabled examination of the orientation of cellulose microfibril deposition in subsidiary cells and in the adjacent epidermal cells as well. In all the species examined the microfibrils of neighbouring cells (both true subsidiary and regular epidermal cells) lying in parallel to the long axis of guard cells had a perpendicular arrangement of microfibrils, as compared to the microfibril orientation of guard-cells (Fig. 3B, D, F, H, J, L). In ferns the microfibrils appeared to encircle the stoma (Fig. 3B, D).

It has long been known that epidermal neighbouring cells can participate in stomatal movements by changes either in turgor or osmotic pressure of the epidermal cells (Stalfelt, 1966). Consequently, the neighbouring cells change their volume and passively open or close the stomata. However, the mechanism of this phenomenon was never fully explored and the underlying cell wall structures are unknown. The parallel arrangement of microfibrils we have observed in the neighbouring cells enables the guard cell to expand outwards while the guard cells shrink.

### Cellulose crystallinity types in stomata

Cellulose microfibrils consist of amorphous and crystalline domains that are further spatially organized into regions of differing crystallinity. Crystalline anisotropic materials are birefringent and can therefore be examined using polarized light microscopy. Retardance, which is an integrated effect of birefringence over a light path, is an approximate measure of crystallinity. Thus, higher retardance values may indicate either higher levels of cellulose crystallinity or the presence of more crystalline cellulose material in the tissue.

**Fig. 4.**
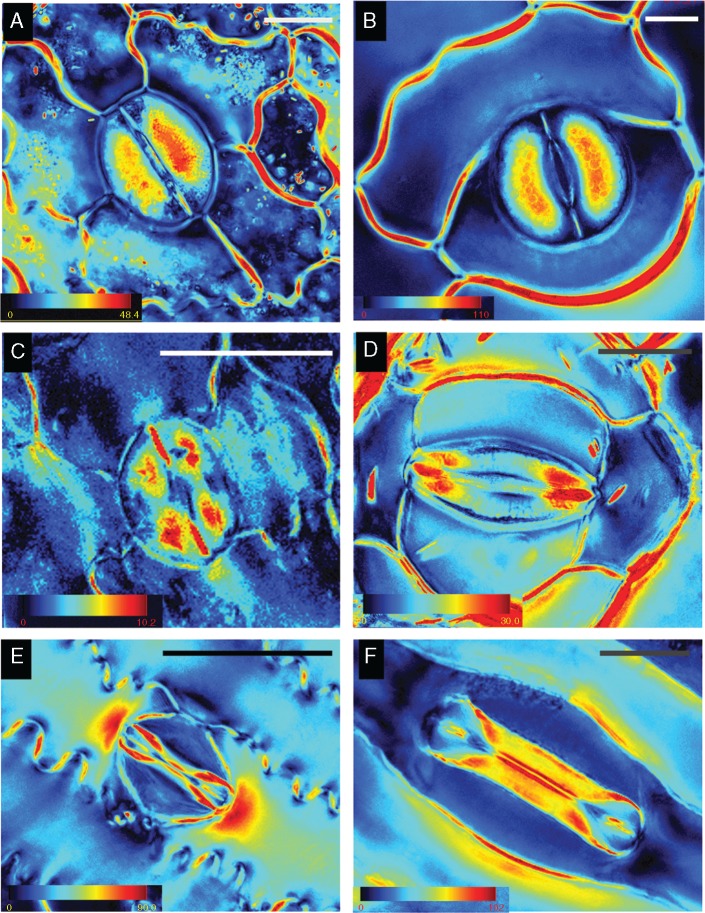
PolScope crystalline cellulose retardance images of stomata. (A) *Asplenium*, (B) *Platycerium*, (C) Arabidopsis, (D) *Commelina* (note the birefringent crystals in the epidermis), (E) *Sorghum*, (F) *Triticum*. The retardance colour scale bar codes the retardance range; note the large differences observed between different species. For polarised light images, see Fig. 3. Scale bars = 20 µm.

We observed three distinct patterns of stomatal retardance, which we classified as Types I, II and III, among the vascular plant species that we examined (Fig. 4, scheme in Fig. 1). Interestingly, despite the conserved orientation pattern of cellulose microfibrils among ferns and angiosperms (Fig. 3B, D, F, H), they displayed completely different patterns of crystalline cellulose deposition. The kidney-shaped stomata of ferns (*Asplenium* and *Platycerium*) exhibited a Type I pattern, with the central region of the guard cell being the most retardant and the polar ends the least retardant (Fig. 4A, B). In contrast, angiosperm kidney-shaped stomata (*Arabidopsis* and *Commelina*) exhibited a Type II pattern, with a less retardant centre, strongly retardant polar end walls and highly retardant areas adjacent to it at the polar ends of the stomatal guard cells (Fig. 4C, D). It is noteworthy that no increased crystallinity was observed along the inner cell wall of the guard cells of the kidney-shaped stomata (Fig. 4A–D). The grasses (*Sorghum* and *Triticum*) exhibited a Type III pattern, with a retardant ventral wall area, and relatively highly retardant periphery of the bulbous polar ends (Fig. 4E, F). *Triticum* stomata showed quite high crystallinity everywhere except the middle and inner portions of bulbous polar ends. It should be noted that we observed these patterns in dozens of stomata for the six species described, as well as in many additional plant species (see Supplementary Data Fig. S2).

**Fig. 5.**
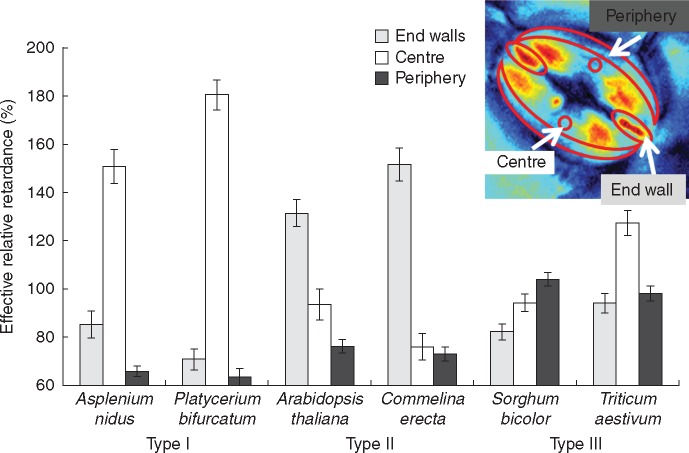
Quantification of relative crystalline cellulose retardance in stomata of various species. Effective retardance of a whole stoma was taken as 100 %, and relative to it, the effective retardance in three different areas was calculated – as seen in the inset.

The absolute retardance values varied greatly between species (see the differences in the retardance scale in Fig. 4), yet the pattern of retardance remained very similar. Therefore, we compared the effective retardance, which was calculated for different areas relative to the total effective retardance of each stoma (Fig. 5). Unsurprisingly, in Type I stomata the central area was the most crystalline, at over 150 %, and in Type II the end-wall was found to be over 120 % crystalline. The highest crystallinity (∼100 %) was associated with the periphery in Type III stomata whereas in direct contrast the lowest crystallinity (∼70 %) was found in the periphery of Type I and II stomata. Interestingly, *Triticum*, possessing a quite crystalline central area, appeared to exhibit a different relative retardance pattern than *Sorghum*.

**Fig. 6.**
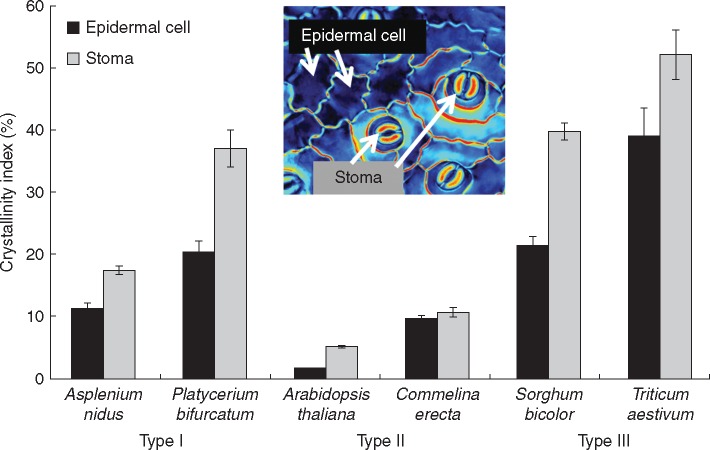
Crystallinity index in stomata and epidermal cells of various species. Retardance was measured in either stoma or epidermal cells using epidermal peels from the abaxial side of the leaf, and crystallinity index was calculated as a percentage of microcrystalline commercial cellulose (Avicel) retardance.

In general, there was considerable variation in crystallinity of stomata and epidermal cells between species. Relative crystallinity index was calculated in comparison to the commercial crystalline cellulose (Avicel) (Fig. 6). In all six species, the stomata had higher crystallinity than the epidermal cells, although this is by no means a universal rule. In *Commelina* the difference was quite small, and in a few additional species, for example *Adianthus* (Fig. S2) stomata were less crystalline than the epidermis. *Arabidopsis* and *Commelina* showed the lowest crystallinity among the six species presented, although this property was also found not to be taxon-specific (see retardance scales for additional species in Fig. S2).

### Lignin and phenolic compounds in stomata

As lignin is a natural fluorochrome, we carried out fluorescence confocal microscopy imaging of lignin. However, because phenolic compounds also fluoresce in the same spectrum, we also used a phloroglucinol staining of lignin (phloroglucinol stains the hydroxycinnamyl aldehyde end-groups in lignins) as a complementary histochemical approach.

**Fig. 7.**
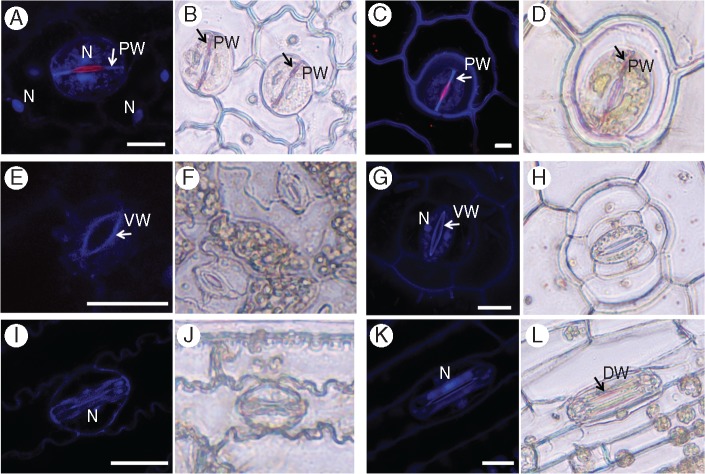
Lignins and phenolic compounds in stomatal guard cells. Lignin (blue) and phenolic compounds (red) autofluorescence was observed in leaf fragments using confocal microscopy (A, C, E, G, I, K) and by phloroglucinol stain in epidermal peels (B, D, F, H, J, L). (A, B) *Asplenium* – note the phenolic compound autofluorescence in the nuclei and red autofluorescence of the ventral wall; (C, D) *Platycerium* – note the red autofluorescence of the ventral wall (white arrow); (E, F) *Arabidopsis*; (G, H) *Commelina*; (I, J) *Sorghum*; (K, L) *Triticum*. N, nucleus; PW, polar end-wall; VW, ventral wall; DW, dorsal wall. Scale bars = 20 µm.

Several different allocation patterns of lignin were apparent. In ferns, the polar walls were positively stained with phloroglucinol (Fig. 7B, D). The staining was distinct when compared to unstained controls (Fig. S3). This correlated well with autofluorescence in ferns, which was also strong at the polar walls (Fig. 7A, C). In *Asplenium* the nuclei also showed a strong autofluorescent signal. Additionally, the ventral walls in both ferns exhibited red autofluorescence (at the chlorophyll spectrum). This red autofluorescence was unlikely to result from the presence of chlorophyll as the leaves were ethanol-bleached prior to imaging. However, other compounds, for example anthocyanins and azulenes, have also been reported to result in red autofluorescence (600–630 nm) of plant tissues (Roshchina, 2008), and further research will be required to determine the specific material that caused the stomatal walls to autofluoresce in the red spectrum.

In *Arabidopsis* and *Commelina* the strongest autofluorescence was observed in the ventral wall, near the stomatal pore (Fig. 7E, G). In *Commelina* the guard cell nuclei were also autofluorescent. In both species no phloroglucinol staining was observed in the guard cells (Fig. 7E, H). In *Commelina* the ventral walls showed red autofluorescence, although it was much weaker than seen in the fern ventral walls (Fig. 7G). No autofluorescence or phloroglucinol staining was observed at the polar ends of *Arabidopsis* and *Commelina* stomata. It is noteworthy that lignin deposition at the polar ends of the fern stomata examined (characteristic of the Type I stomata in the current study) overlaps with the area of high crystalline cellulose deposition in angiosperms (representing the Type II stomata). In the grasses a strong autofluorescence signal was observed in ventral walls and in the whole stoma in general (Fig. 7I, K). However, no phloroglucinol staining was observed for *Sorghum* stomata and it was very weak in dorsal walls of *Triticum* stomata (Fig. 7J, L).

### Pectins in stomata

**Fig. 8.**
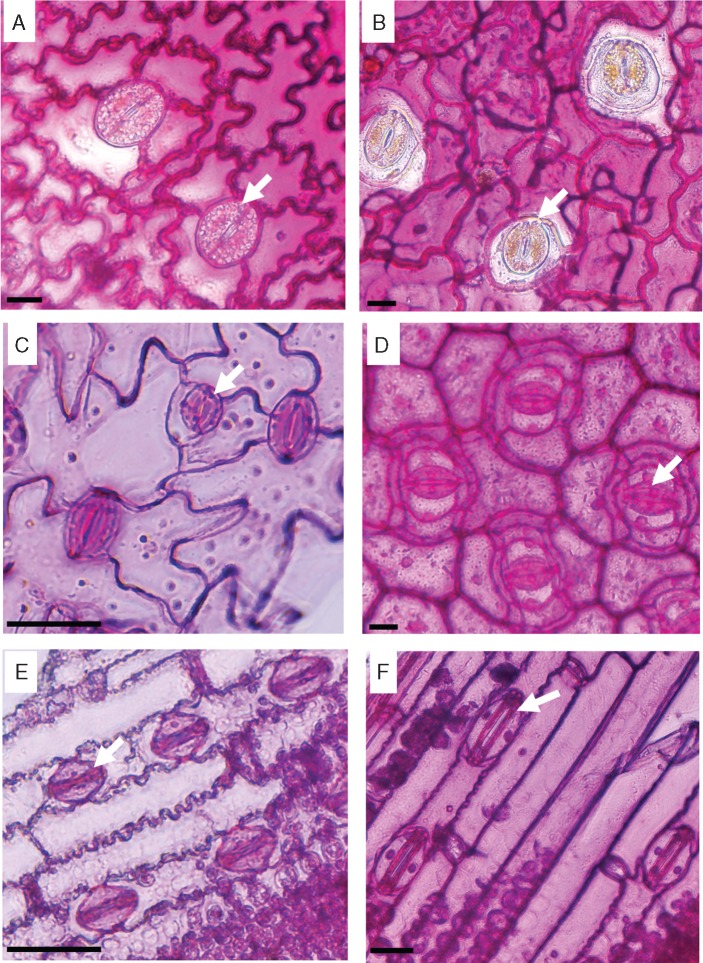
Epidermal peels stained with ruthenium red for pectins. (A) *Asplenium*, (B) *Platycerium*, (C) *Arabidopsis*, (D) *Commelina*, (E) *Sorghum*, (F) *Triticum*. Scale bars = 20 µm. Arrows indicate stomata.

Pectin staining of epidermal peels, with ruthenium red, showed large differences between the ferns and the angiosperms (Fig. 8). The fern guard cell walls had less pectins compared with those of the surrounding epidermal cells (Fig. 8A, B), while in the angiosperms the guards cell walls were strongly stained compared with the surrounding cells (Fig. 8C–F).

### Numerical biomechanical simulations

**Fig. 9.**
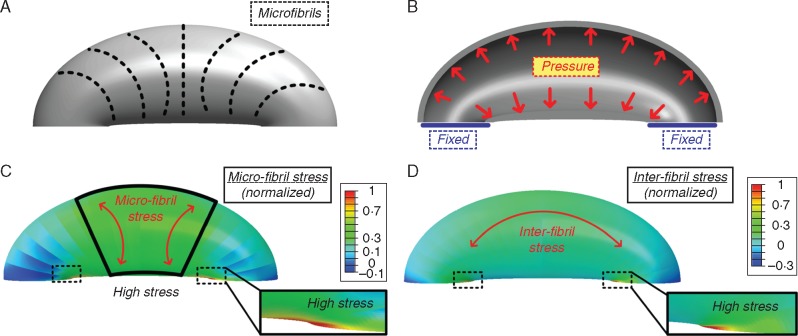
Stoma structural model used for the numerical simulations (A, B) and the resulting finite-elements numerical simulation (C, D). (a) Schematic description of the localized circumferential micro-fibril directionality in external view. (B) The simulations boundary conditions, fixed edge displacement and uniform internal pressure in cross-sectional view. (C) Normalized microfibril stress field; high microfibril stresses are obtained at the middle of the stoma and at the edges. Red arrows indicate the microfibril stress direction. (D) Inter-fibril stress field; high inter-fibril stresses are obtained at the stoma edges. Red arrow indicates the inter-fibril stress direction.

Numerical mechanical simulations were used to identify possible origins for the localized lignification and crystallinity modification found within the stoma structure (Fig. 9). The numerical simulations revealed the microfibril stress field around the stoma (Fig. 9C) and the inter-fibril stress field along the stoma (Fig. 9D) on the stoma surface. It can be seen that a large region of high microfibril stress is obtained at the centre of the stoma surface, while additional localized stress enhancement, both in the fibril and in the inter-fibril directions, is obtained at the polar end-walls. The former effect can be correlated with the circumferential deformation confinement, which is essential for pore opening (Aylor *et al.*, 1973), while the latter possibly emerges from reaction forces between the guard cells enclosing the pore.

## DISCUSSION

Stomata are widely considered to have evolved only once and first appeared about 400 million years ago, before xylem, leaves, seeds or flowers (Beerling and Franks, 2009). They remain a key attribute of plant function and, remarkably, various stomatal features including the mechanisms that regulate stomatal movement (Chater *et al.*, 2011; Ruszala *et al.*, 2011), numerous stomatal genes (Ruszala *et al.*, 2011) and morphology are among the few plant features that have remained relatively unchanged throughout millennia. However, as the climate changed, atmospheric CO_2_ and O_2_ concentrations, water availability and temperature fluctuated, new taxa emerged and consequently guard cell wall structure has continuously adapted to specific environmental challenges. When the first stomata appeared, the CO_2_ concentration on Earth was about ten times higher than its present value (Royer *et al.*, 2004), enabling easy CO_2_ uptake even in plants without stomata (Raven, 2002). This supports suggestions that the earliest stomata functioned as drying pores for the sporophyte before spore release (Duckett *et al.*, 2009), and only later acquired their current function in gas exchange. Fluctuations in atmospheric CO_2_ concentration correspond with the appearance of major plant groups (Beerling *et al.*, 2001; Haworth *et al.*, 2011), and very likely also drove stomatal evolution. Several studies have suggested that early diverging land plants, including extant mosses and ferns, together with cycads and gymnosperms are less sensitive to CO_2_ concentration than flowering plants (Brodribb *et al.*, 2009; Field *et al.*, 2015) although this is controversial and disputed by some researchers (Ruszala *et al.*, 2011; Franks and Britton-Harper, 2016). Indeed it would be interesting to investigate and compare the CO_2_ sensitivity of polypod ferns that diverged after the emergence of flowering plants and the decline in atmospheric CO_2,_ with the earlier evolving fern groups and flowering plants. Ambient atmospheric CO_2_ concentration has declined within the last several million years, while the lower end of this range is marginal for C_3_ plants (Robinson, 1994). Thus, Robinson (1994) hypothesized that declining CO_2_ concentration imposed a physiological strain on plants and this constraint drew the development of superior stomatal efficiency in grasses. This evolutionary context should be kept in mind when examining the mechanical functioning of externally similar-looking stomata. 

This research attempted to integrate structural data, phylogenetic parameters and biomechanical modelling to investigate the functional properties of stomatal cell walls. Our data demonstrate for the first time the existence of distinct spatial patterns of varying cellulose crystallinity in guard cell walls. We observed three distinct types of stomatal cell wall crystallinity (Types I, II and III) that were delimited to different taxonomic groups (Figs 1 and4); additional types may exist in other species. As far as we know, this is also the first time that such structural heterogeneity of cellulose crystallinity has been shown in the same cell (the layered structure of fibre cells is probably the closest example, although there the cellulose crystallinity is homogenously distributed throughout each layer). Those crystallinity patterns could serve two possible purposes: either (1) locally increasing stiffness and load-bearing, or (2) a means of differentially binding other cell wall components. For instance, the non-crystalline (amorphous) cellulose regions more readily absorb water (Chami Khazraji and Robert, 2013) and bind xyloglucans and pectins (Zykwinska *et al.*, 2005). Such local functional differences between crystalline and amorphous cellulose regions could offer exciting possibilities in the precise control and optimization of cell wall function as a part of the mechanism employed in stomata opening/closing.

While kidney-shaped stomata have a preserved morphology, they showed different patterns of crystallinity and phenolics as well as differences in deposition of lignin and pectins between ferns and angiosperms. Subsequently, the numerical simulations indicated two high-stress regions in the surface of the cell walls of kidney-shaped stomata: at the centre of the stoma in the microfibril direction, and at the polar end-walls both in the microfibril and in the inter-fibril directions (Fig. 9C, D). To attenuate possible damage, localized material modifications are required in the high-stress regions. Type I (fern) stomata indeed possess a significantly higher cellulose crystallinity at the centre stoma region, and locally lignified polar end-walls; from a mechanical perspective both modifications locally increase the stiffness and strength of the cell wall material. In Type II (kidney-shape angiosperms) stomata, the lignified edges are replaced by a localized enhancement of the crystallinity of cellulose microfibrils; both modifications produce equivalent mechanical effects which strengthen the stoma edges from potential damage. It has yet to be determined whether there are additional cell wall components/modifications providing stiffness in the centre of the stoma region of angiosperms. According to Ziegler (1987), after lignin and lignification appeared in Pteridophyta, lignin remained generously used in pteridophytes and gymnosperms, whereas it is more sparingly used in the more recent angiosperm lineage. It is intriguing that in angiosperms crystalline cellulose might play a similar role to lignin in stomatal end-walls, and could reflect differences in evolutionary pressures at the time that the lineages evolved. It is possible that the polypod ferns, which are a large monophyletic group (Schneider *et al.*, 2004) that evolved after the emergence of flowering plants, are unusual in the occurrence of high levels of lignin in their guard cell walls. This could be further investigated by studying the guard cell wall composition of a wider selection of ferns, including the leptosporangiate ferns, non-polypod eusporangiate ferns and gymnosperms.

While the relatively high crystallinity in the centre of the fern stomata corresponds with the high stress in the same region shown by the numerical simulation, the angiosperm kidney-shaped stomata lack this region of increased crystallinity. This indicates basic underlying differences in cell wall structure between ferns and angiosperms. Our results show that while angiosperm stomata are rich in pectins, this is not the case with ferns (Fig. 8). Also, although the dumbbell-shaped stomata of grasses had a different cellulose crystallinity pattern, they were pectin-rich as with kidney-shaped angiosperms (Fig. 8E, F). Stomata in many plant species have abundant pectins (Ziegler, 1987), and pectins are known to be important for the stomatal movement mechanism in several angiosperm species (Jones *et al.*, 2003, 2005). Several studies have shown that pectins have a strong impact on cell wall stiffness and, correspondingly, elasticity. The moss *Funaria* has abundant pectins present in the guard cell walls during the early stages of their development. However, at later developmental stages pectin content is reduced and coincides with the loss of flexibility (Merced and Renzaglia, 2014). Pectin degradation causes tissue softening in *Solanum* pollen tubes (Parre and Geitmann, 2005) and ripening fruits (Brummell, 2006). Pectins were linked to increased elasticity of spruce needles (Renault and Zwiazek, 1997) and in thistle flowers (Marga *et al.*, 2003). It has been proposed that pectins have a load-bearing role (Peaucelle *et al.*, 2012), not unlike the cellulose, and possibly can compensate for cellulose deficiency (Aouar *et al.*, 2010). We suspect that pectins in angiosperm stomata serve a load-bearing function: ferns use crystalline cellulose as a localized strengthening material in the central region, whereas in angiosperms pectins may serve a similar role.

Our results demonstrate several additional differences in stomatal cell wall constituents between the phylogenetic groups. Stomata showed different UV autofluorescence patterns (Fig. 7), being found at the polar end-walls in ferns, near the pore in the kidney-shaped angiosperm stomata and over the entire guard cell in grasses. Stomatal autofluorescence in response to UV excitation has been noted previously (Hutzler *et al.*, 1998; Yuan *et al.*, 2013) and was attributed to lignin, phenolics and ferulic acid. Similar patterns of stomatal autofluorescence were seen by Jones *et al.* (2005). In the kidney-shaped stomata of the angiosperms *Commelina communis* and *Vicia faba* fluorescence was strongest at the ventral wall near the pore, and in the grass *Zea mays* it was quite strong throughout the guard cell, with a stronger signal at the dorsal wall. The authors attributed the fluorescent signal to ferulic acid esters. Interestingly, phenolic cell wall constituents were implicated in cell wall hardening (Fan *et al.*, 2006). In addition, fern inner ventral walls showed red autofluorescence, which was not caused by chlorophyll or anthocyanins, as those had been ethanol-extracted prior to examination. This autofluorescence may be attributed to azulenes, which have been found, for instance, in the cell walls of *Equisetum arvense* spores (Roshchina *et al.*, 2002).

Intriguingly, the three distinct guard cell wall types we demonstrate in this study might be related to the three cell wall types reported in land plants. Eudicots and many monocots have xyloglucan and pectin-rich Type I walls, commelinid monocots possess arabinoxylans rich and pectin low Type II walls, while many ferns have mannan-rich and pectin low Type III walls (Carpita and Gibeaut, 1993; Silva *et al.*, 2011). However, in our study *Commelina* (a commelinid monocot) had a similar guard cell wall composition to the dicotyledon Arabidopsis, while the grasses (also commelinids) guard cell walls exhibited a different wall type. It is important to bear in mind that the designation of plant cell wall Types I–III is based on material derived from all the cells present in the plant rather than for specific cells and that the specific composition of particular cell types may differ substantially from the predominant cell type present. Furthermore, it is likely that the composition of cell walls of highly specialized cells and tissues evolved under a different set of restraints than the majority of the cell types present in a plant. Therefore, we prefer to remain cautious about the comparison of the known cell wall types with the guard cell types described in our study. Undoubtedly, much more research of plant cell wall composition, particularly at the cellular and tissue levels, must be conducted on a broad evolutionary array of plant species to settle the numerous unanswered questions.

To conclude, although the current study was conducted on only six plant species, our results suggest a more general phenomenon. The different stomatal cell wall attributes we investigated (cellulose crystallinity, pectins, lignin, phenolics) exhibit clear taxon-specific patterns, with reciprocal substitution of structural elements. These differences may reflect modifications to the stomatal complex that occurred in response to specific environmental challenges and that have allowed stomata to retain their distinct structure without compromising function.

## AUTHOR CONTRIBUTIONS

I.S., S.H. and B.B. planned and designed the research. I.S., Y.S. and Z.M. performed experiments. I.S., B.B., Z.P. and S.H. wrote the manuscript. Z.P. and A.S. contributed to the experimental design and data interpretation.

## SUPPLEMENTARY DATA

Supplementary data are available online at https://academic.oup.com/aob and consist of the following. Data S1: mechanical modelling and finite-elements simulations. Figure S1: SEM images of stomata of (a) *Asplenium*, (b) *Platycerium*, (c) *Arabidopsis*, (d) *Commelina*, (e) *Sorghum* and (f) *Triticum*. Note the thick ventral cell walls. S, stoma; SC, subsidiary cell. Figure S2: PolScope crystalline cellulose retardance images of stomata of (a) *Nephrolepis* – fern, (b) *Adianthus* – fern, (c) *Nymphaea* – core angiosperm, (d) *Cyclamen persicum* – dicotyledon, (e) *Vicia faba* – dicotyledon and (f) *Cyperus papyrus* – sedge (Poales). Retardance scale colour codes the retardance range; note the large differences between species. Scale bars = 20 µm. Figure S3: lignins and phenolic compounds in stomata: autofluorescence using confocal microscopy (a,b) and lignin staining (c,d). (a) Bright light and (b) autofluorescence image of *Commelina* stoma. *Asplenium* stoma either unstained (c) or stained (d) with phloroglucinol for lignin. Scale bars = 20 µm.

## Supplementary Material

Supplementary Data
