# Supplementary material for: Stomatal cell wall composition: distinctive structural patterns associated with different phylogenetic groups
Source: Ann Bot. 2017 Jan 31;119(6):1021–33. doi: 10.1093/aob/mcw275 (PMC5604698; doi:10.1093/aob/mcw275)
Supplement: Supplementary Data [file mcw275_Supp.zip › aob-16430-s04.docx]

**Data S1: Mechanical modeling and FE simulations**

The FE simulations employed linear-elastic material with anisotropic mechanical properties in the circumferential vs. perpendicular directions. Customary models from composite materials mechanics were applied to estimate the moduli anisotropy of the stomata; in particular the circumferential and longitudinal moduli were evaluated by the Voigt and Reuss models, respectively (i.e. Direct and Inverse Rule-Of-Mixtures) - $E_{C}=E_{f}\phi_{f}+E_{m}\left( 1-\phi_{f} \right)$, and $E_{L}=1/\left[ \phi_{f}/E_{f}+\left( 1-\phi_{f} \right)/E_{m} \right]$. For evaluating the stomata moduli, typical characteristics of plant tissues were employed (Gibson 2012), specifically $E_{f}=130GPa$ for the Young’s modulus of the micro-fibrils, $E_{m}=5GPa$ for the hemicellulose matrix and micro-fibril volume fraction of $\phi_{f}=0.2$. By incorporating these typical parameters into the above models, a modulus ratio of $E_{C}/E_{L}\sim1:5$ is obtained for the stomata anisotropy.

Sensitivity analysis was conducted via a set of axillary simulations, taking into account additional moduli ratios (1:3 and 1:10) that approximately represent the typical upper and lower values for the moduli ratio – obtained by incorporating the typical data rang for the cellulose micro-fibrils and hemicellulose from the literature into the above models. These simulations produced negligible changes at normalized stress morphologies from the results for the moduli ratio 1:5, and are thus not shown (for compactness).
