# Supplementary figures and images for: Stomatal cell wall composition: distinctive structural patterns associated with different phylogenetic groups
Source: Ann Bot. 2017 Jan 31;119(6):1021–33. doi: 10.1093/aob/mcw275 (PMC5604698; doi:10.1093/aob/mcw275)

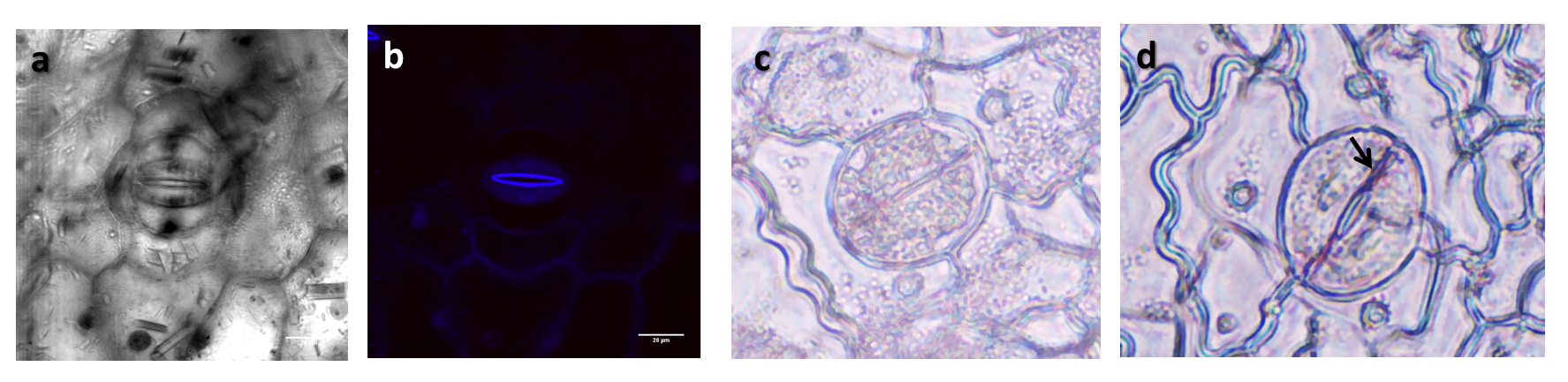

Supplement: Supplementary Data [file mcw275_Supp.zip › aob-16430-s03.tif]

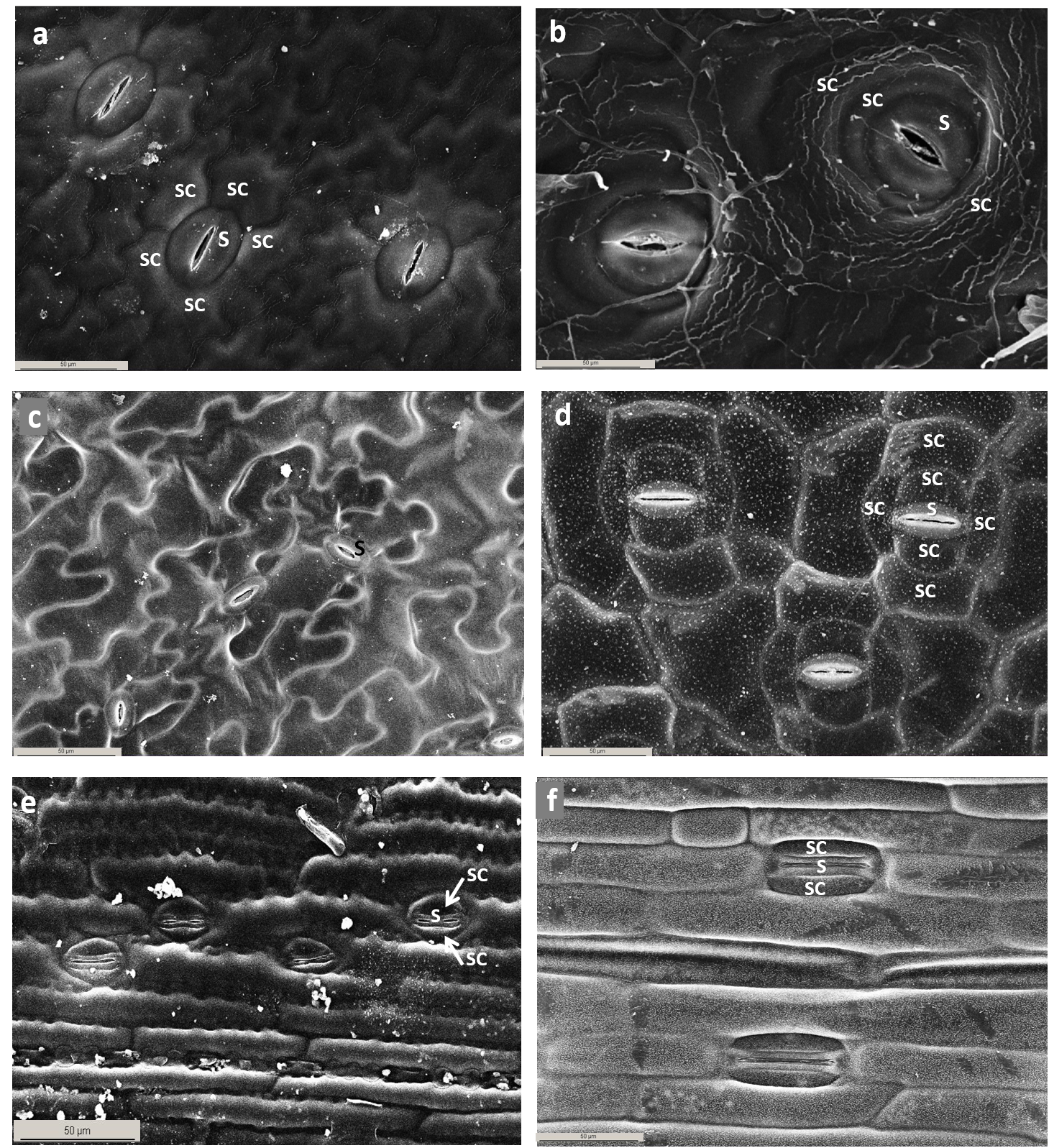

Supplement: Supplementary Data [file mcw275_Supp.zip › aob-16430-s01.tif]

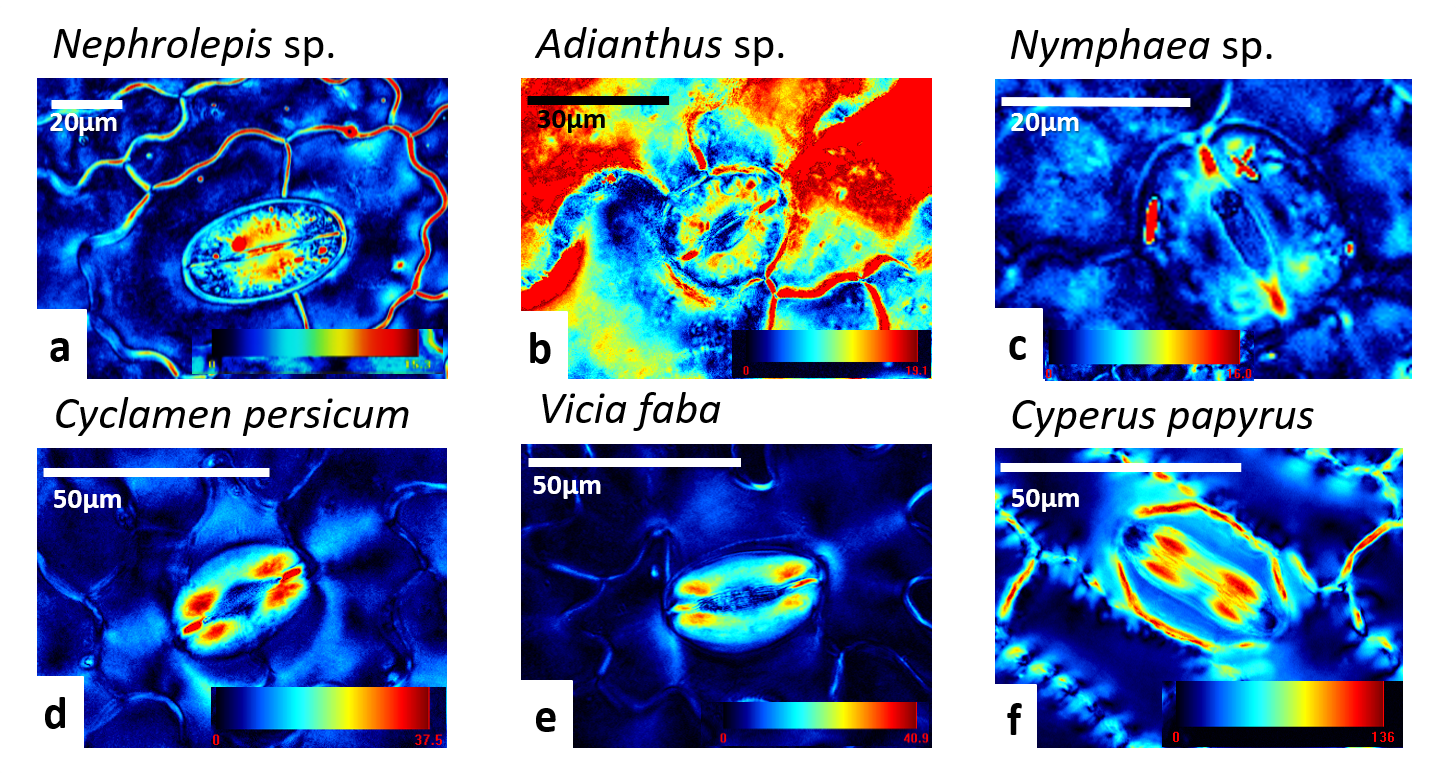

Supplement: Supplementary Data [file mcw275_Supp.zip › aob-16430-s02.tif]
